# Supplementary material for: Chiral and flat-band magnetic quasiparticles in ferromagnetic and metallic kagome layers
Source: Nat Commun. 2024 Feb 21;15:1592. doi: 10.1038/s41467-024-45841-8 (PMC10882050; doi:10.1038/s41467-024-45841-8)
Supplement: Supplementary file 1 — Supplementary Information [file 41467_2024_45841_MOESM1_ESM.pdf]

Supplementary Information: Chiral and flat-band magnetic quasiparticles in ferromagnetic and metallic kagome layers

S. X. M. Riberolles,[1] Tyler J. Slade,[1] Tianxiong Han,[1,2] Bing Li,[1] D. L. Abernathy,[3]
P. C. Canfield,[1,2] B. G. Ueland,[1] P. P. Orth,[1,2] Liqin Ke,[1] and R. J. McQueeney[1,2]

[1]*Ames Laboratory, Ames, IA, 50011, USA*

[2]*Department of Physics and Astronomy,*
Iowa State University, Ames, IA, 50011, USA

[3]*Neutron Scattering Division, Oak Ridge National Laboratory, Oak Ridge, TN 37831 USA*

(Dated: January 16, 2024)

EXPERIMENTAL DETAILS

$TbMn_6Sn_6$ crystallizes in the $HfFe_6Ge_6$-type structure with hexagonal space group P6/mmm (No. 191) and lattice parameters a and c are 5.530 and 9.023 Å at 300 K [S1].

HEISENBERG MODEL DESCRIPTION

The Heisenberg model is given by $\mathcal{H} = \mathcal{H}_{\text{intra}} + \mathcal{H}_{\text{inter}} + \mathcal{H}_{\text{aniso}}$. Each Mn kagome layer possesses strong NN FM exchange ($\mathcal{J}_1 = -28.8$ meV),

$$\mathcal{H}_{\text{intra}} = \mathcal{J}_1 \sum_{\langle i<j \rangle} \mathbf{s}_i \cdot \mathbf{s}_j \tag{S1}$$

where s is the Mn spin operator with magnitude $s = 1$. Several unique interlayer magnetic couplings between Mn layers and between Mn and Tb layers are found, giving

$$\mathcal{H}_{\text{inter}} = \sum_k \sum_{i<j} \mathcal{J}_k^{MM} \mathbf{s}_i \cdot \mathbf{s}_{j+k} + \mathcal{J}^{MT} \sum_{\langle i<j \rangle} \mathbf{s}_i \cdot \mathbf{S}_j. \tag{S2}$$

Here, $\mathcal{J}^{MT} = 0.93$ meV is the AF coupling between neighboring Mn and Tb layers, with Tb having a total spin angular momentum of $S = 3$ and Mn having $s = 1$. Competing interactions between Mn layers with a layer index k up to $k = 3$ ($\mathcal{J}_1^{MM} = -4.4$ meV, $\mathcal{J}_2^{MM} = -19.2$ meV, $\mathcal{J}_3^{MM} = 1.8$ meV) are necessary to fully describe the low energy spin waves, especially the splitting of AO and AE modes. Finally, uniaxial and easy-plane single-ion anisotropies for Tb and Mn, respectively, are described by

$$\mathcal{H}_{\text{aniso}} = K^T \sum_i (S_i^z)^2 + K^M \sum_i (s_i^z)^2 \tag{S3}$$

with $K^T = -1.28$ meV and $K^M = +0.44$ meV [S2, S3].

Extended models where longer-range intralayer Mn-Mn interactions have been introduced, such as J_2, J_{3a} (through the Mn atom), and J_{3b} (across the hexagon). Generally, longer-range FM interactions pull down the average energy OE and OO modes in better correspondence to experiments. We try not to affect the AO and AE modes by introducing constraints. For example, keeping $J_1 + 3J_2 = constant$ will keep the M-point energies of the AE and AO modes fixed. Fig. S1 shows some results for the extended models.

In Fig. S2, we show constant energy slices from the Heisenberg model parameters used in the main text. This can be compared to the data shown in Fig. 2 of the main text. The model

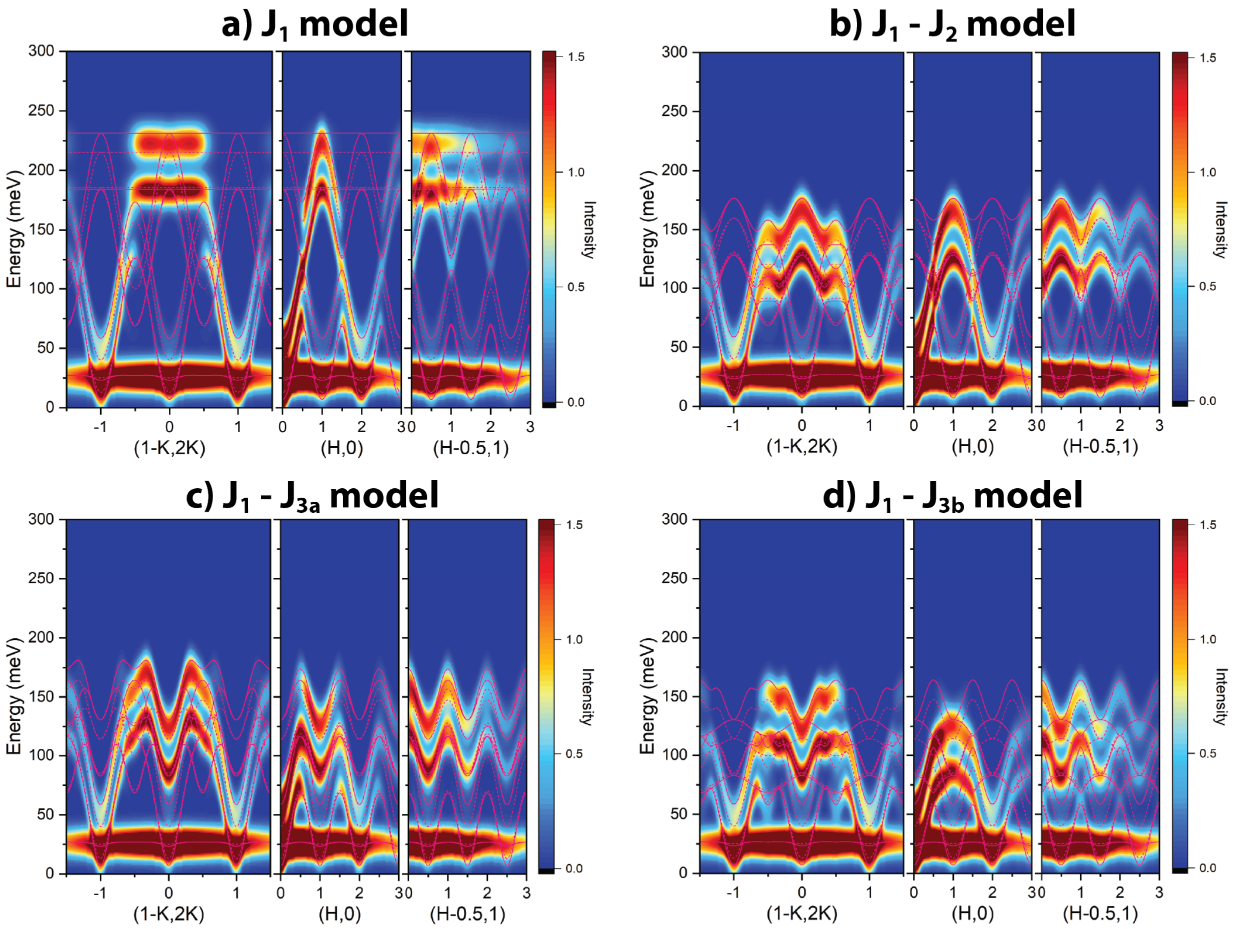

FIG. S1. Calculations of the neutron scattering intensity along the (1-K,2K), (H,0) and (H-0.5,1) directions for Heisenberg model with (a) Nearest-neighbor intralayer interaction $J_1 = -28.8$ meV, (b) nearest and next-nearest neighbor interactions with $J_1 = -15$ meV and $J_2 = -4.6$ meV and $J_1 + 3J_2 = -28.8$ meV, (c) nearest and third-nearest neighbor (through the Mn) interactions with $J_1 = -12$ meV and $J_{3a} = -8.4$ meV and $J_1 + 2J_{3a} = -28.8$ meV, (d) nearest and third-nearest neighbor (across the hexagon) interactions with $J_1 = -12$ meV and $J_{3b} = -8.4$ meV and $J_1 + 2J_{3b} = -28.8$ meV.

calculations indicate that intensity principally occurs in the Γ zones below 130 meV (comprised of AE and AO modes) and only in the Γ' zones above 130 meV (comprised of OE,OO,FE, and FO modes). The flat band modes that occur at the highest energies have intensities centered at the K-point.

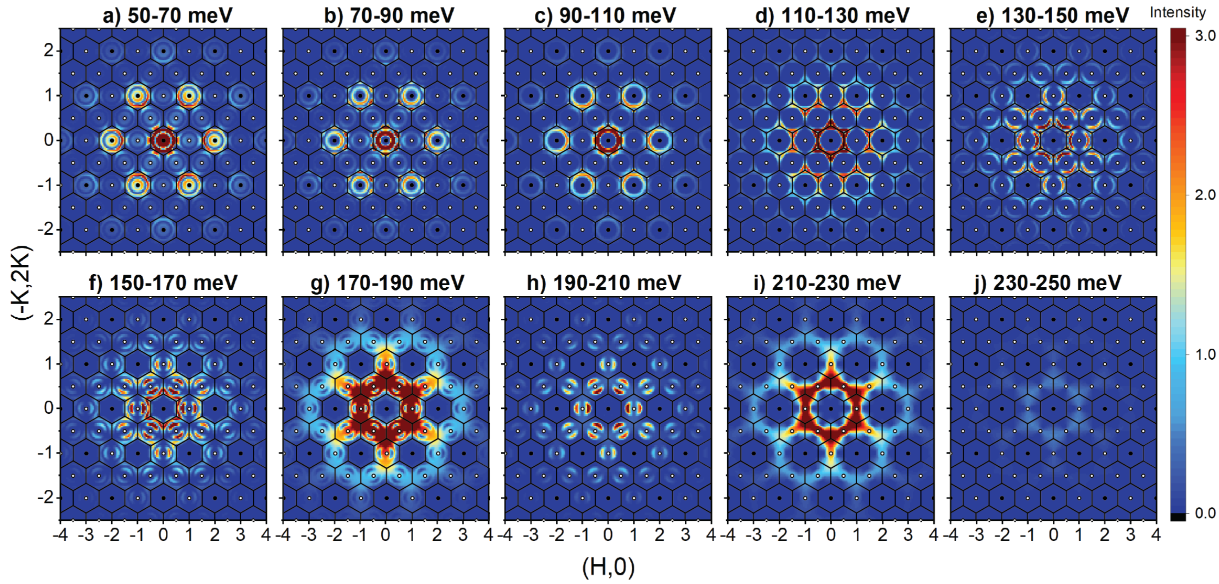

FIG. S2. Constant energy slices of the $TbMn_6Sn_6$ spin excitations from a Heisenberg model. Slices are identical to the data cuts shown in Fig. 2 and are made over incremental energy ranges from (a) 50–70 meV, (b) 70–90 meV, (c) 90–110 meV, (d) 110–130 meV, (e) 130–150 meV, (f) 150–170 meV, (g) 170–190 meV, (h) 190–210 meV, (i) 210–230 meV, (j) 230–250 meV. Data were collected at $E_i = 250$ meV for panels (a)–(e) and $E_i = 500$ meV for panels (f)–(j). All cuts are averaged over an L-range from -7 to 7 rlu. Hexagonal Brillouin zone boundaries are shown and Γ and Γ' zone centers are indicated by filled and empty circles, respectively.

DAMPED HARMONIC OSCILLATOR ANALYSIS

Figure 1(i) in the main text shows cuts at the key reciprocal space positions for the chiral and flat band excitations. Here, we fit the imaginary part of the dynamical magnetic susceptibility to a to a damped harmonic oscillator lineshape, $\chi''(E) = A\gamma E/[(E^2 - \omega_i^2)^2 + E^2\gamma_i^2]$, plus a background. We find the mean frequencies of the chiral and flat mode cluster excitations, ω_C =145(2) meV and ω_F =187(8) meV, and the corresponding relaxation rates, γ_C =69(10) meV and γ_F =115(30) meV, respectively. The relaxation rates are influenced by the background fitting, so we used the cut at $\Gamma = (2, 0)$ to estimate the high-energy background level. Nonetheless, an oscillator Q-factor of $\omega/\gamma \approx 1.5 - 2$ for the two modes is consistent with large damping.

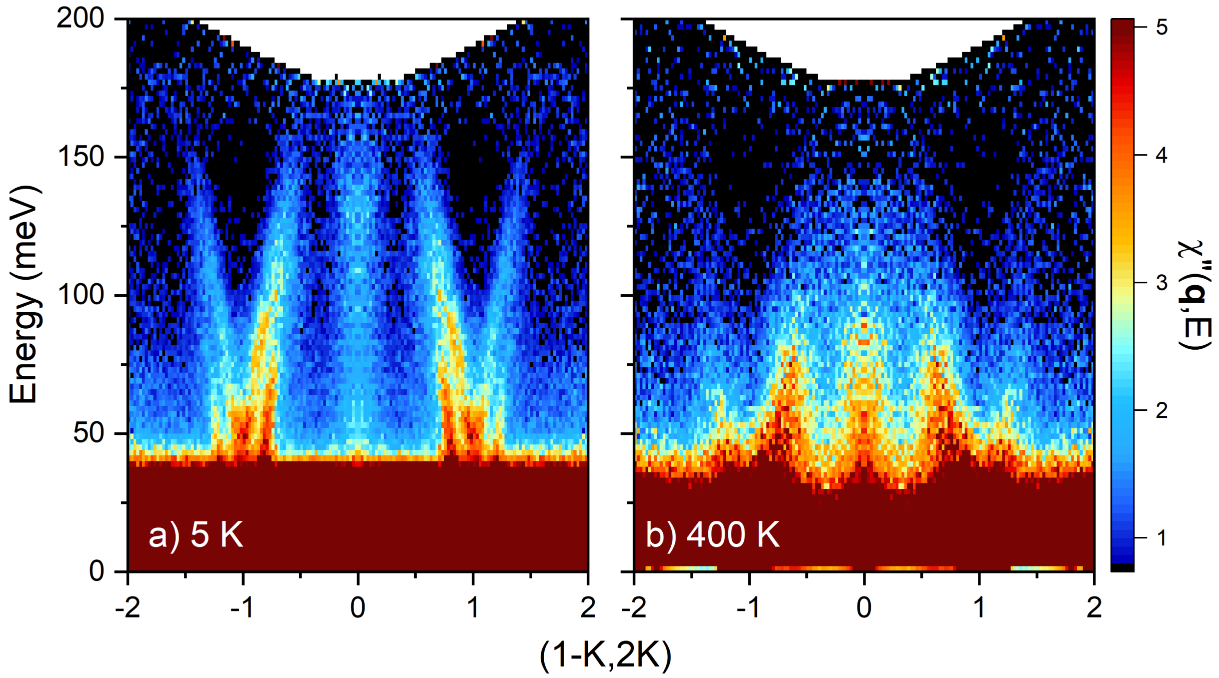

FIG. S3. Comparison of slices along the $(-K, 2K, 0)$ direction for a) $T = 5$ K and b) 400 K. Data are proportional to the dynamical susceptibility, $\chi''(\mathbf{q}, E) = I(\mathbf{q}, E)(1 - \exp(-E/kT))$. Cuts are performed with $E_i = 250$ meV. All plots are also averaged over reciprocal space ranges of $H = [0.9 : 1.1]$ and $L = [-7 : 7]$. Data are symmetrized according to the $P6/mmm$ space group to improve statistics.

TEMPERATURE DEPENDENCE

Figure S3 compares slices through excitations in the Γ and Γ' zones at $T = 5$ K and 400 K as measured with $E_i = 250$ meV for several energy transfers. These data show that the dispersive AO and AE excitations in the (-1,2,0) Γ zone are further split at 400 K, indicating significant softening of the Mn modes just below T_C. The localized $q = 0$ cluster mode in the (1,0,0) Γ' zone is still observed at 400 K. Both the dispersive Γ and localized Γ' modes are more intense at lower energies, but their intensity rapidly diminishes above 100 meV as a consequence of softening and mode damping near T_C. Evidence of the dispersive Γ mode is present at 130 meV. However, the Γ' excitation appears to be completely suppressed above 130 meV at 400 K. Despite the complex temperature dependence, which also must take into account the transition from uniaxial to easy-plane ferrimagnetism above $T_{SR} = 310$ K, the data suggest that the both the Γ and Γ' excitations occur on an ordered Mn sublattice.

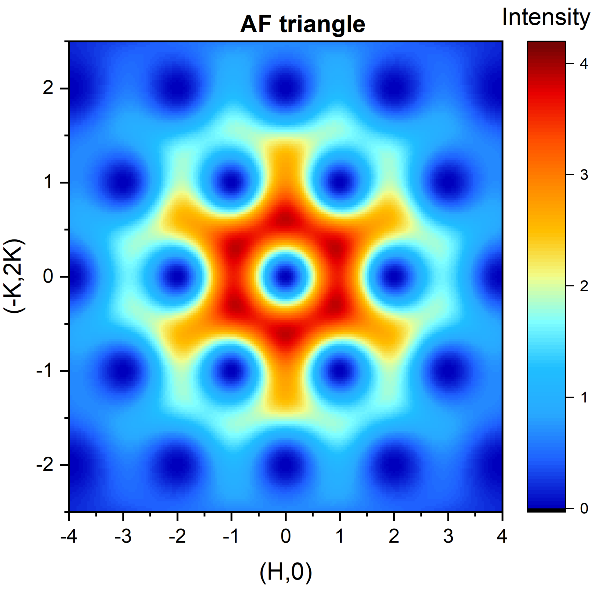

FIG. S4. Calculation of the structure factor of a triangular cluster with chiral Γ' spin correlations.

PLAQUETTE STRUCTURE-FACTOR CALCULATIONS

Triangular plaquette. Spin correlations are most likely to occur in hexagonal or trangular plaquettes in the kagome layer. In Fig. S4, we plot the structure factor for chiral spin correlations around a triangular plaquette. This pattern is not consistent with the q-dependence of the data in the Γ' zones shown in Fig. 2(a)–(e). Fig. 3(g) shows a schematic diagram of flat band and chiral spin excitations which have no phase coherence from hexagon-to-hexagon.

Chiral spin correlations with planar magnetization. Above 310 K, $TbMn_6Sn_6$ adopts a planar magnetization. In this scenario, chiral spin correlations originating from spin fluctuations that are transverse to the magnetization (with vector chirality pointing in the plane) will involve and in-plane and out-of-plane components of the instantaneous moment. To account for the planar magnetization and compare to the uniaxial case, we must also average the neutron intensity over the three different domains of the planar magnetization vector. The neutron intensity for an arbitrary direction of the magnetization is given by the sum

$$S(\mathbf{q}) = f^2(q) \sum_{\alpha\beta} (\delta_{\alpha\beta} - \hat{q}_\alpha \hat{q}_\beta) S_{\alpha\beta} \tag{S4}$$

and

$$S_{\alpha\beta}(\mathbf{q}) = \sum_{ij} S^i_\alpha (S^j_\beta)^* \exp[\mathbf{q} \cdot (\mathbf{r}^j - \mathbf{r}^i)] \tag{S5}$$

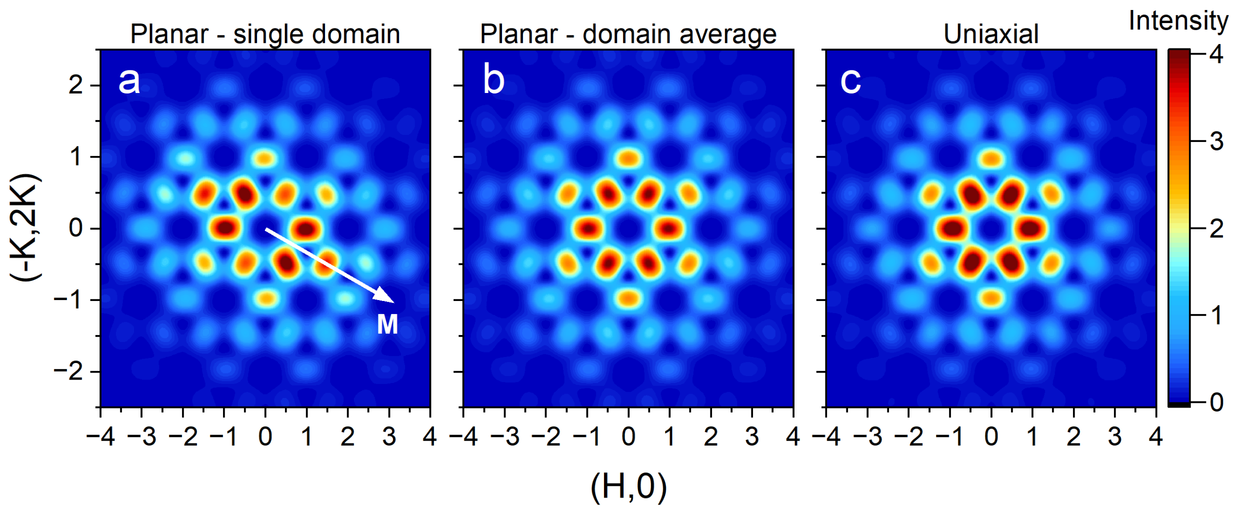

FIG. S5. (a) Calculation of the structure factor for transverse chiral spin correlations of a single-domain planar magnetization with magnetization direction (and vector chirality) pointing along the x direction (shown as the white arrow labeled "M"). (b) Chiral structure factor for planar magnetization after averaging over all three domains. (c) Comparison to the chiral structure factor for uniaxial magnetization pointing along z.

where S_α are the spin components transverse to the magnetization.

For example, if the planar magnetization points along the x direction, then transverse chiral spin correlations have components $S_x = 0$, $S_y^j = \sin\phi_j$, and $S_z^j = \cos\phi_j$. When suitably averaging over all instantaneous configurations and L, then Fig. S5(a) shows that a single planar domain gives a chiral scattering pattern with in-plane intensity anisotropy consistent with the average magnetization direction. After averaging over all three equivalent planar magnetization domains, the planar chiral structure factor (Fig. S5(b)) is identical to the uniaxial structure factor (Fig. S5(c) to within an overall scale factor. The scale factor depends on the data analysis choices, such as the L-averaging. In general, these results indicate that the unpolarized neutron scattering data is unable to determine the direction of the vector chirality after domain averaging.

MASSIVE DIRAC FERMIONS

Figure S6, shows the expectation for intravalley and intervalley charge scattering for massive Dirac fermions (MDFs), which form a continuum of scattering near the Γ and K-points. Experimental data place the chemical potential at $E_D = -130$ meV with a band gap of $\Delta \approx 30$ meV [S4]. The features of the continuum of scattering from interband transitions of the MDFs show similar

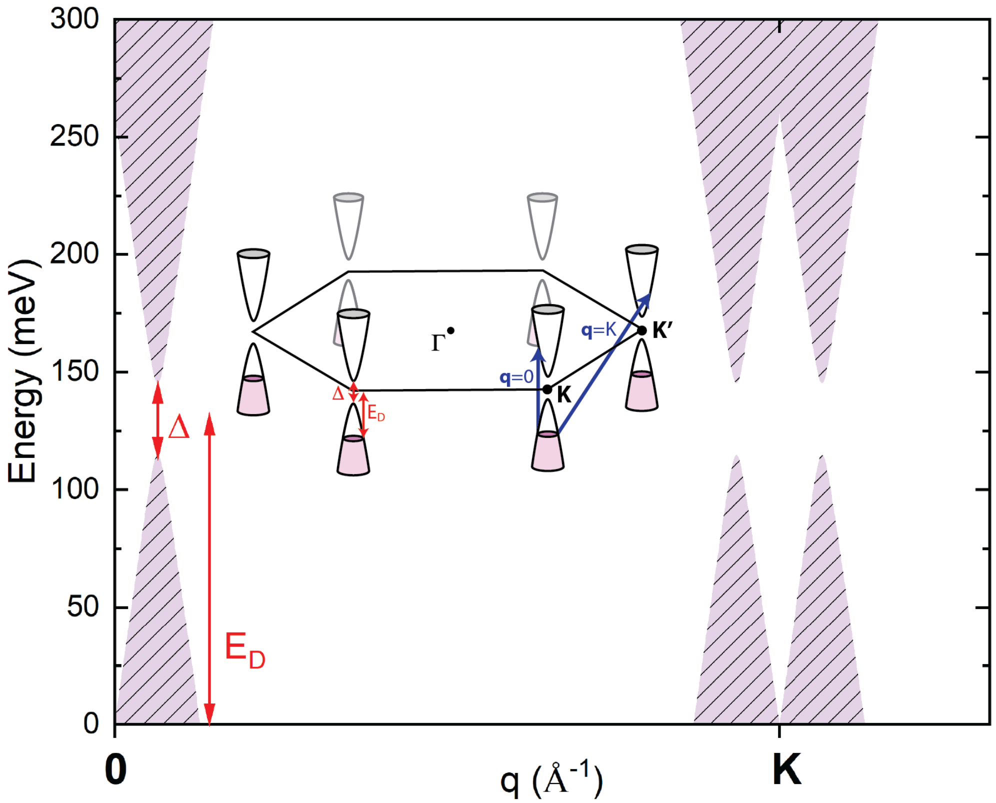

FIG. S6. Continuum of electron-hole scattering channels for massive Dirac fermions in a 2D kagome layer. Dirac fermions are characterized by the chemical potential E_D and gap Δ.

energy onset and q-dependence as the data. However, the spin-polarized nature of the MDFs indicates that these are not spin-flip transitions and therefore should not couple to magnons. This assumption is supported by our DFT calculations.

DENSITY-FUNCTIONAL THEORY CALCULATIONS AND LANDAU DAMPING

Band structure. The electronic structures of $TbMn_6Sn_6$ were calculated in the DFT+U approximation using an all-electron full-potential LMTO (FP-LMTO) [S5]. Plain DFT usually incorrectly positions the Lanthanide 4f states near the Fermi level. A sizable on-site Hubbard $U \approx 7$ eV correction was applied on Tb-f states to push down the occupied $4f$ states and further away from the Fermi level. The resulting spin magnetic moments and band structure compare well with those previously calculated using other full-potential all-electron methods [S6].

Susceptibility calculation. After obtaining the self-consistent *ab initio* Hamiltonian H, we calculate the bare transverse spin susceptibility $\chi_0^{+-}(\mathbf{q}, \omega)$ using the eigenvalues and eigenfunctions

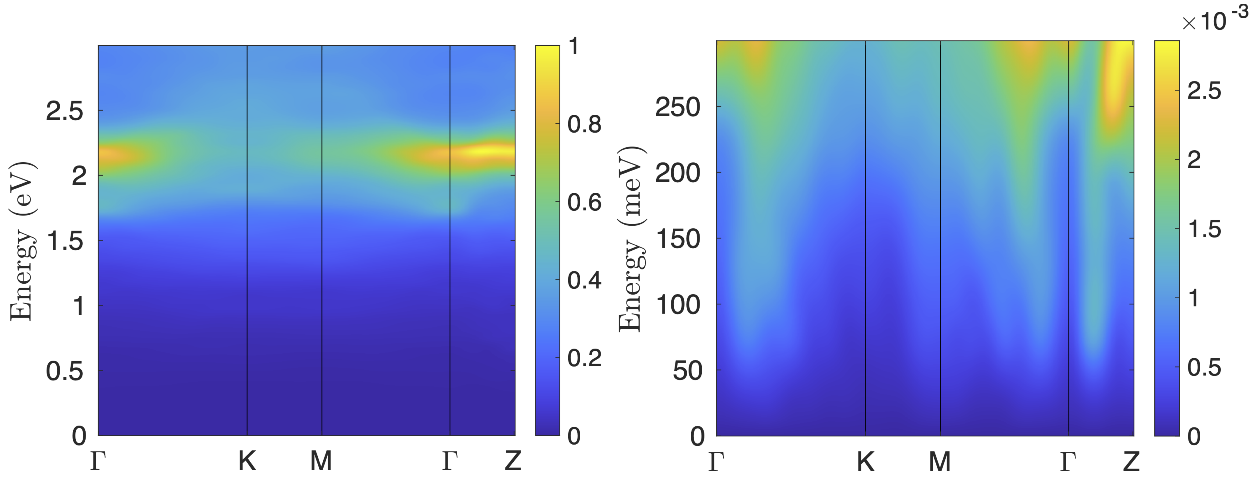

FIG. S7. The imaginary part of the bare transverse spin susceptibility $\mathrm{Im}[\chi_0^{+-}(\mathbf{q},\omega)]$ along the high symmetry path Γ–K–M–Γ–Z in $TbMn_6Sn_6$. The intensity of $\mathrm{Im}[\chi_0^{+-}(\mathbf{q},\omega)]$, normalized with $\max(\mathrm{Im}[\chi_0^{+-}(\mathbf{q},\omega)])$, is shown in the energy windows of 0–3 eV (left panel) and 0–300 meV (right panel), respectively. $\mathrm{Im}[\chi_0^{+-}(\mathbf{q},\omega)]$ characterizes the intensity of single-particle spin-flip excitations—the Stoner excitations.

of H within a linear response theory [S7–S10],

$$\begin{aligned}\chi_0^{+-}(\mathbf{r},\mathbf{r}',\mathbf{q},\omega) &= \sum_{\mathbf{k}n\downarrow}^{\mathrm{occ}}\sum_{\mathbf{k}'n'\uparrow}^{\mathrm{unocc}} \frac{\Psi^*_{\mathbf{k}n\downarrow}(\mathbf{r})\Psi_{\mathbf{k}'n'\uparrow}(\mathbf{r})\Psi^*_{\mathbf{k}'n'\uparrow}(\mathbf{r}')\Psi_{\mathbf{k}n\downarrow}(\mathbf{r}')}{\omega-(\epsilon_{\mathbf{k}'n'\uparrow}-\epsilon_{\mathbf{k}n\downarrow})+i\delta} \\ &+ \sum_{\mathbf{k}n\downarrow}^{\mathrm{unocc}}\sum_{\mathbf{k}'n'\uparrow}^{\mathrm{occ}} \frac{\Psi^*_{\mathbf{k}n\downarrow}(\mathbf{r})\Psi_{\mathbf{k}'n'\uparrow}(\mathbf{r})\Psi^*_{\mathbf{k}'n'\uparrow}(\mathbf{r}')\Psi_{\mathbf{k}n\downarrow}(\mathbf{r}')}{-\omega-(\epsilon_{\mathbf{k}n\downarrow}-\epsilon_{\mathbf{k}'n'\uparrow})+i\delta},\end{aligned} \tag{S6}$$

where $\mathbf{k}'=\mathbf{q}+\mathbf{k}$. For computational efficiency, χ_0 was calculated using a mixed basis [S11], which consists of the product basis [S11, S12] within the augmentation spheres and interstitial plane waves. We first calculate $\chi_0^{+-}(\mathbf{q},\omega)$ on a $12\times12\times6$ $\mathbf{q}$ mesh and construct the $\chi_0^{+-}(\mathbf{R},\omega)$ through Fourier transformation. Then we use the resulting $\chi_0^{+-}(\mathbf{R},\omega)$ to calculate $\chi_0^{+-}(\mathbf{q},\omega)$ with a dense set of $\mathbf{q}$ points along high-symmetry paths.

The Mn-d bands dominate near the Fermi level, governing the spin-flip excitations. Figure S7 shows the imaginary part of the bare transverse spin susceptibility $\mathrm{Im}[\chi_0^{+-}(\mathbf{q},\omega)]$ along the high symmetry path Γ–K–M–Γ–Z. As shown in the left panel, the most prominent Stoner excitations occur near 2.2 eV, corresponding to the exchange splitting of Mn-3d bands that dominate near the Fermi level. However, since $TbMn_6Sn_6$ is a metallic system, small finite $\mathbf{q}$-dependent $\chi_0^{+-}(\mathbf{q},\omega)$ can also be observed in the energy window of magnon excitations (right panel), which may cause the magnon damping.

[S1] B. Malaman, et al., ”Magnetic properties of RMn_6Sn_6 (R=Gd-Er) compounds from neutron diffraction and Mössbauer measurements”, J. Magn. Magn. Mater.**202**, 519-534 (1999).

[S2] S. X. M. Riberolles, Tyler J. Slade, D. L. Abernathy, G. E. Granroth, Bing Li, Y. Lee, P. C. Canfield, B. G. Ueland, Liqin Ke, R. J. McQueeney, ”Low-Temperature Competing Magnetic Energy Scales in the Topological Ferrimagnet $TbMn_6Sn_6$”, Phys. Rev. X, **12**, 021043 (2022).

[S3] S. X. M. Riberolles, Tyler J. Slade, R. L. Dally, P. M. Sarte, Bing Li, Tianxiong Han, H. Lane, C. Stock, H. Bhandari, N. J. Ghimire, D. L. Abernathy, P. C. Canfield, J. W. Lynn, B. G. Ueland, and R. J. McQueeney, ”Orbital character of the spin-reorientation transition in $TbMn_6Sn_6$”, Nat. Comm., **14**, 2658 (2023).

[S4] W. Ma, X. Xu, J.-X. Yin, H. Yang, H. Zhou, Z.-J. Cheng, Y. Huang, Z. Qu, F. Wang, M. Z. Hasan et al., “Rare Earth Engineering in RMn$_6$Sn$_6$ (R =Gd-Tm,Lu) Topological Kagome Magnets”, Phys. Rev. Lett. **126**, 246602 (2021).

[S5] M. Methfessel, M. van Schilfgaarde, and R. A. Casali, Chapter 3 a full-potential LMTO method based on smooth Hankel functions, in Electronic Structure and Physical Properties of Solids: The Uses of the LMTO Method, Lecture Notes in Physics, Vol. 535, edited by H. Dreysse (Springer-Verlag, Berlin, 2000).

[S6] Y. Lee, R. Skomski, X. Wang, P. P. Orth, A. K. Pathak, B. N. Harmon, R. J. McQueeney, I. I. Mazin, and L. Ke, “Interplay between magnetism and band topology in Kagome magnets RMn_6Sn_6”, arXiv:2201.11265 (2022).

[S7] T. Kotani and M. van Schilfgaarde, “Spin wave dispersion based on the quasiparticle self-consistent GW method: NiO, MnO and α-MnAs”, J. Phys.: Condens. Matter **20**, 295214 (2008).

[S8] L. Ke and M. I. Katsnelson, “Electron correlation effects on exchange interactions and spin excitations in 2D van der Waals materials”, npj Comp. Mater. **7**, 1 (2021).

[S9] B. Li, D. M. Pajerowski, S. X. M. Riberolles, L. Ke, J.-Q. Yan, and R. J. McQueeney, “Quasi-two-dimensional ferromagnetism and anisotropic interlayer couplings in the magnetic topological insulator $MnBi_2Te_4$”, Phys. Rev. B **104**, L220402 (2021).

[S10] L. Ke, M. van Schilfgaarde, J. Pulikkotil, T. Kotani, and V. Antropov, “Low-energy coherent stoner-like excitations in $CaFe_2As_2$”, Phys. Rev. B: Rapid Commun. **83**, 060404 (2011).

[S11] T. Kotani, M. van Schilfgaarde, and S. V. Faleev, “Quasiparticle self-consistent gw method: A basis

for the independent-particle approximation", Phys. Rev. B **76**, 165106 (2007).

[S12] F. Aryasetiawan and O. Gunnarsson, "Product-basis method for calculating dielectric matrices", Phys. Rev. B **49**, 16214 (1994).
